# Preclinical evaluation of ADVM-062, a novel intravitreal gene therapy vector for the treatment of blue cone monochromacy

**DOI:** 10.1016/j.ymthe.2023.03.011

**Published:** 2023-03-16

**Authors:** Kelly Hanna, Julio Nieves, Christine Dowd, Kristina Oresic Bender, Pallavi Sharma, Baljit Singh, Mark Renz, James N. Ver Hoeve, Diana Cepeda, Claire M. Gelfman, Brigit E. Riley, Ruslan N. Grishanin

**Affiliations:** 1 Adverum Biotechnologies, Inc., Redwood City, CA 94063, USA; 2 Ocular Services on Demand (OSOD), Madison, WI 53719, USA

**Keywords:** blue cone monochromatism, AAV, gene therapy, intravitreal, OPN1LW, non-human primate, animal models, cone photoreceptors, fovea, inherited retinal disorder

## Abstract

Blue cone monochromacy (BCM) is a rare X-linked retinal disease characterized by the absence of L- and M-opsin in cone photoreceptors, considered a potential gene therapy candidate. However, most experimental ocular gene therapies utilize subretinal vector injection which would pose a risk to the fragile central retinal structure of BCM patients. Here we describe the use of ADVM-062, a vector optimized for cone-specific expression of human L-opsin and administered using a single intravitreal (IVT) injection. Pharmacological activity of ADVM-062 was established in gerbils, whose cone-rich retina naturally lacks L-opsin. A single IVT administration dose of ADVM-062 effectively transduced gerbil cone photoreceptors and produced a *de novo* response to long-wavelength stimuli. To identify potential first-in-human doses we evaluated ADVM-062 in non-human primates. Cone-specific expression of ADVM-062 in primates was confirmed using ADVM-062.myc, a vector engineered with the same regulatory elements as ADVM-062. Enumeration of human OPN1LW.myc-positive cones demonstrated that doses ≥3 × 10^10^ vg/eye resulted in transduction of 18%–85% of foveal cones. A Good Laboratory Practice (GLP) toxicology study established that IVT administration of ADVM-062 was well tolerated at doses that could potentially achieve clinically meaningful effect, thus supporting the potential of ADVM-062 as a one-time IVT gene therapy for BCM.

## Graphical abstract

**Figure:**
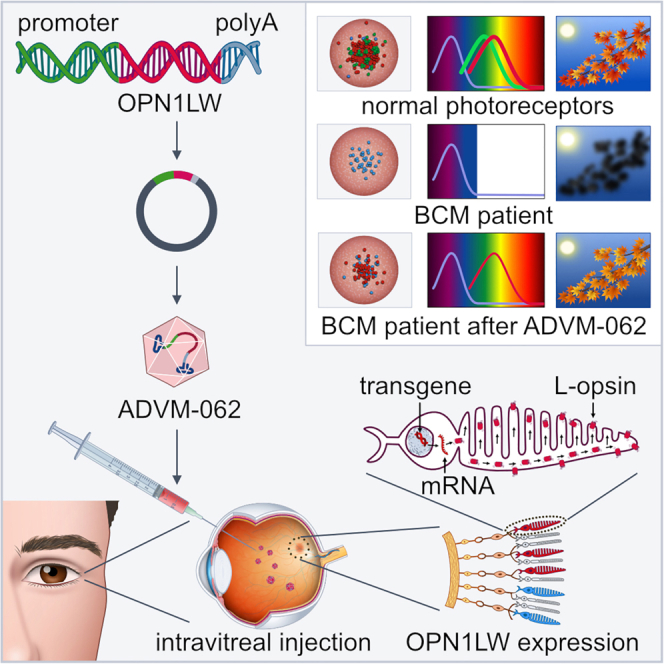

## Teaser

Hanna and colleagues have developed ADVM-062, an intravitreal AAV.7m8 ocular gene therapy for blue cone monochromacy designed to deliver human L-opsin to cone photoreceptors. The study demonstrates meaningful foveal cone transduction in NHPs at well-tolerated doses, thus supporting potential clinical development of ADVM-062 as a gene therapy for BCM patients.

## Introduction

Blue cone monochromacy (BCM) is a rare X-linked retinal disease resulting from the congenital absence of L- and M-opsin proteins. BCM is characterized by severely impaired color discrimination, poor visual acuity, nystagmus, and photophobia.^1^ The disease results either from large deletions involving upstream regulatory sequences such as in the locus control region (LCR; a long-range *cis*-regulatory genomic element that controls cone-specific and mutually exclusive expression of the L- and M-opsins) or from other structural changes in the *OPN1LW/OPN1MW* gene cluster. Some of the structural changes in the gene cluster arise from non-homologous recombination between the L- and the M-opsin genes followed by a missense point mutation inactivating the residual gene or nonsense mutation, resulting in premature termination and nonsense-mediated mRNA decay, or from rare haplotypes in exon 3 of the cone opsin mRNA that result in aberrant splicing.^1^^,^^2^^,^^3^^,^^4^^,^^5^^,^^6^^,^^7^^,^^8^^,^^9^^,^^10^^,^^11^ L and M cones are the principal photoreceptors of the fovea centralis, wherein the fovea contains the highest density of L and M cones, which have primary roles in high-acuity foveal vision. The loss of both L- and M-opsins results in decreased visual acuity ranging from 20/63 to 20/200, with most patients assessed as partly sighted and ∼8%–10% patients as legally blind, and significant deficiencies in color discrimination.^12^^,^^13^ The light aversion is often reported by patients as debilitating, with severe migraines and the inability to lead a normal life during daylight hours.^13^ At present, there are no approved therapies for BCM available. Current management includes the use of refractive corrective lenses, tinted lenses for disabling photophobia, and educational support. Delivery of either human M-opsin or human L-opsin to cones by means of gene therapy has the potential to restore foveal function, alleviate other debilitating BCM manifestations, and significantly improve quality of life of BCM patients. While BCM is considered a stationary disease, some patients experience progressive cone loss and retinal thinning and atrophy, potentially driven by specific disease-causing mutations in BCM.^13^^,^^14^^,^^15^^,^^16^ Indeed, the cone loss in some BCM patients could limit the therapeutic opportunity for visual restoration by gene therapy, particularly at later stages of the disease, thus signifying the potential importance of fovea imaging and cone photoreceptor topography analysis in candidates for therapy.^13^ Patients with disease that has progressed to overt macular atrophy may benefit from induced pluripotent stem cell (iPSC)-derived retinal photoreceptor precursor cells, which have been demonstrated to differentiate into cones in the non-human primate (NHP) model of retinal degeneration.^17^ Studies in animals support the delivery of functional copies of visual opsins to cone photoreceptors using adeno-associated virus (AAV)-based gene therapy. Studies in dichromatic male New World monkeys demonstrated functional expression of L-opsin in the retina using subretinal AAV2/5 delivery.^18^ The feasibility of restoring cone outer-segment structure and function by gene therapy has been demonstrated by studies in a genetic mouse model of BCM that lacks M-opsin or both M- and S-opsins.^19^^,^^20^^,^^21^ However, the subretinal delivery utilized in the studies described above is poorly suited to BCM patients. This injection route may lead to damage of the fovea/central macula, the primary retinal target region for treating BCM, which may be particularly fragile in this patient population.^12^^,^^13^^,^^22^ To circumvent this problem, ADVM-062 (AAV.7m8-MNTC-hOPN1LW), a novel AAV vector for cone-specific expression of human L-opsin optimized for intravitreal (IVT) delivery, was developed. ADVM-062 is based on a variant of AAV serotype 2, AAV.7m8, which has been shown to efficiently target foveal cones following IVT administration in NHPs.^23^^,^^24^^,^^25^ To ensure cone-restricted expression, ADVM-062 was developed to contain a novel and tissue-specific set of expression elements—MNTC—which contain regulatory sequences from the human L/M-opsin locus, including the LCR and a minimal M-opsin promoter.^26^^,^^27^ We evaluated ADVM-062 in two animal models, Mongolian gerbils and cynomolgus monkeys. The studies established that IVT injection of ADVM-062 resulted in the expression of functionally active L-opsin in cone photoreceptors and demonstrated that IVT-administered ADVM-062 resulted in the effective transduction and cone-restricted expression of human L-opsin in the fovea. A Good Laboratory Practice (GLP) toxicology study established that IVT administration of ADVM-062 was well tolerated at doses that can potentially achieve clinically meaningful functional outcomes, supporting the potential of ADVM-062 as a one-time IVT gene therapy for BCM.

## Results

### Vector characterization

**Figure 1:**
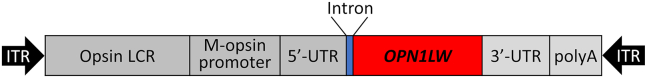
ADVM-062 is an intravitreal gene therapy designed to deliver the human L-opsin gene to the cone photoreceptors The human L-opsin cassette includes a human LCR enhancer sequence, a truncated M-opsin promoter sequence, and a 5′ untranslated region (5′ UTR) based on the M-opsin 5′ UTR, with an inserted chimeric intron and *OPN1LW* coding sequence. An M-opsin 3′ UTR and SV40 polyadenylation sequence were placed after the L-opsin cDNA. The cassette is flanked by AAV2 inverted terminal repeats (ITRs). The OPN1LW expression cassette is packaged in the AAV.7m8 capsid variant. The nucleotide sequence of ADVM-062 is available from patent WO2022204594.^27^

ADVM-062 is a recombinant AAV (rAAV)-based gene therapy vector packaged in the AAV.7m8 variant and designed to express human L-opsin protein under the control of the synthetic cone cell-specific expression cassette (MNTC).^26^^,^^27^ The MNTC regulatory cassette was selected for its ability to drive robust protein expression in M- and L-cone photoreceptors. The cassette included a core LCR enhancer sequence from the human L- and M-opsin genomic locus and a minimal sequence from the human M-opsin gene. In addition, the cassette included a 5′ untranslated region (5′ UTR) based on the M-opsin 5′ UTR, modified to have minimal secondary structure and to include an additional sequence at its 3′ end into which a chimeric intron was inserted. The human L-opsin cDNA sequence was followed by a 3′ UTR that corresponds to the downstream genomic region of M-opsin (Figure 1).^27^ The cone specificity and efficiency of the AAV.7m8 capsid combined with MNTC regulatory elements was initially characterized in African green monkeys using the surrogate vector AAV.7m8-MNTC-GFP, containing regulatory elements identical to those of ADVM-062 but with an EGFP reporter as a transgene. The NHP model was essential due to its retinal anatomy that highly resembles that of the human retina.^28^ Animals were administered IVT AAV.7m8-MNTC-GFP (5 × 10^11^ vector genomes [vg]/eye). A scanning laser ophthalmoscopy (SLO) study of the topology of EGFP expression in eyes showed efficient transduction of the fovea and peripheral retina (Figure S1A), in agreement with earlier studies of the regional distribution of transduction by AAV.7m8-based vectors.^23^^,^^25^ Evaluation of the cellular specificity of EGFP expression demonstrated robust, cone-specific expression of EGFP in the fovea and peripheral retina, shown by the colocalization of EGFP with L/M-opsin and calbindin (used as a marker of peripheral and perifoveal cones) (Figure S1B).^29^

**Figure 2:**
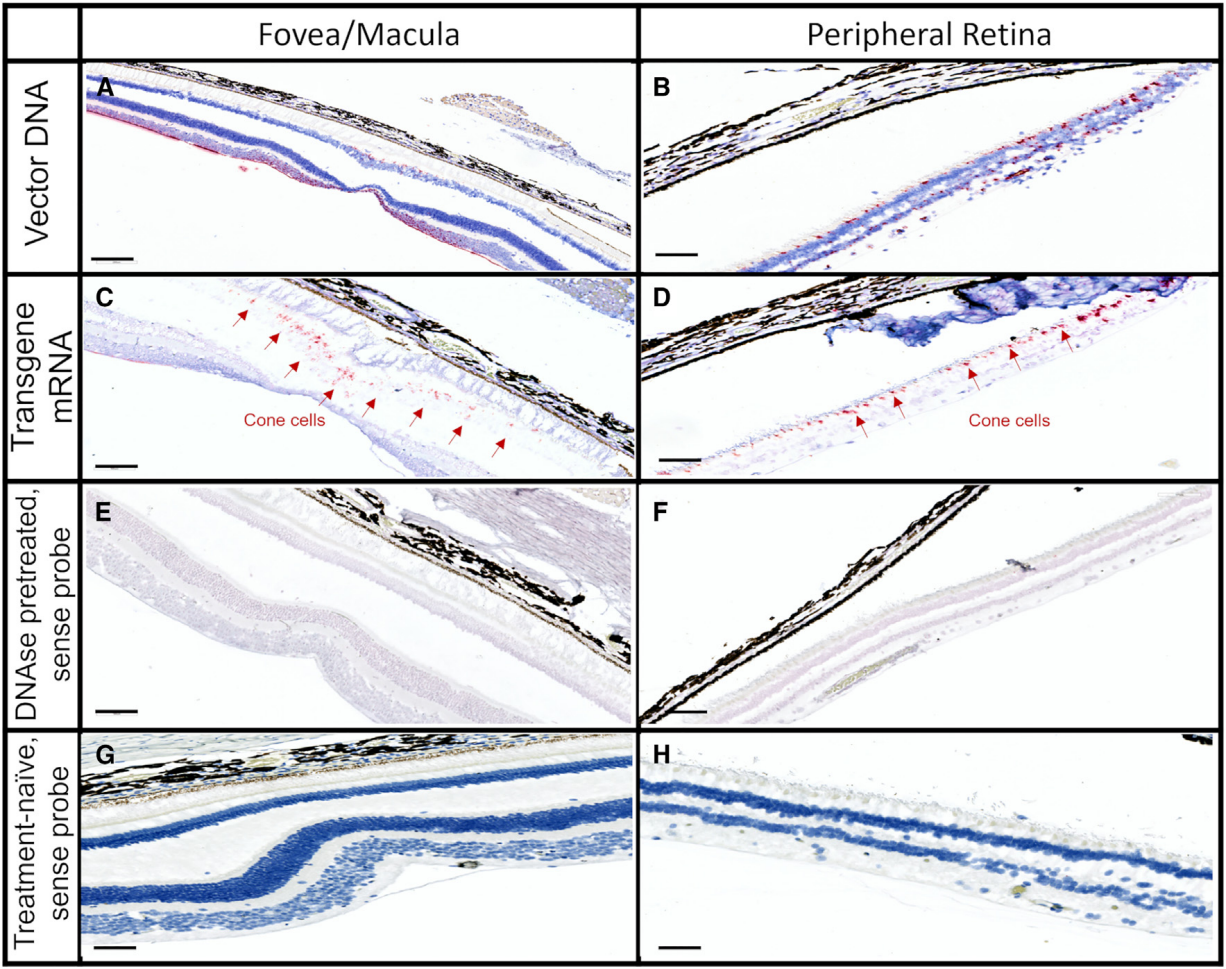
In situ hybridization detection of vector genome DNA and transgene mRNA in the NHP eye administered ADVM-062 IVT at 2 × 10^12^ vg/eye Localization of vector genome DNA in retinal ganglion cells, inner nuclear cells, and photoreceptors of the macula (A) and peripheral retina (B). Localization of h-L-opsin transgene mRNA was detected exclusively in cone cells by using antisense probe after DNase I pretreatment in macula (C) and peripheral retina (D). Localization of transgene was exclusively observed in cone photoreceptor cells in the fovea/macula and peripheral retina. Staining with sense probe after DNase I pretreatment was negative in macula (E) and peripheral retina (F). Staining with sense probe in sections from naive NHP was negative in macula (G) and peripheral retina (H). Red, ADVM-062 DNA/mRNA; blue, nuclei. Scale bars represent 200 μm.

After confirming the expected cone photoreceptor specificity of the MNTC regulatory elements in our vector, NHPs (*Macaca fascicularis*) were dosed with our lead ADVM-062 candidate at 2 × 10^12^ vg/eye. Eight weeks following IVT administration of ADVM-062, the distribution of the AAV vector genome and transgene expression were analyzed on a single-cell basis by *in situ* hybridization (ISH) (BaseScope technology). As depicted in Figure 2, MNTC-driven cone-specific expression of human L-opsin from ADVM-062 was confirmed by detection of L-opsin transgene mRNA in retinal tissue. Evaluation of the retinal presence of ADVM-062 vector genomes by sense and antisense probes specific to vector and to a transgene (Table S1) but not to NHP sequences revealed the wide distribution of vector DNA in the inner limiting membrane, retinal ganglion cell, and inner nuclear layers of the macula/fovea and peripheral retina (Figures 2A and 2B). Unmasking the L-opsin mRNA signal by pretreatment with DNase in the NHP eye with a subsequent hybridization with antisense probes revealed transgene mRNA expression localized exclusively in cone photoreceptor cells in the fovea and peripheral retina, in agreement with the data obtained with AAV.7m8-MNTC-GFP (Figures 2C and 2D).

### Pharmacological activity of ADVM-062

**Figure 3:**
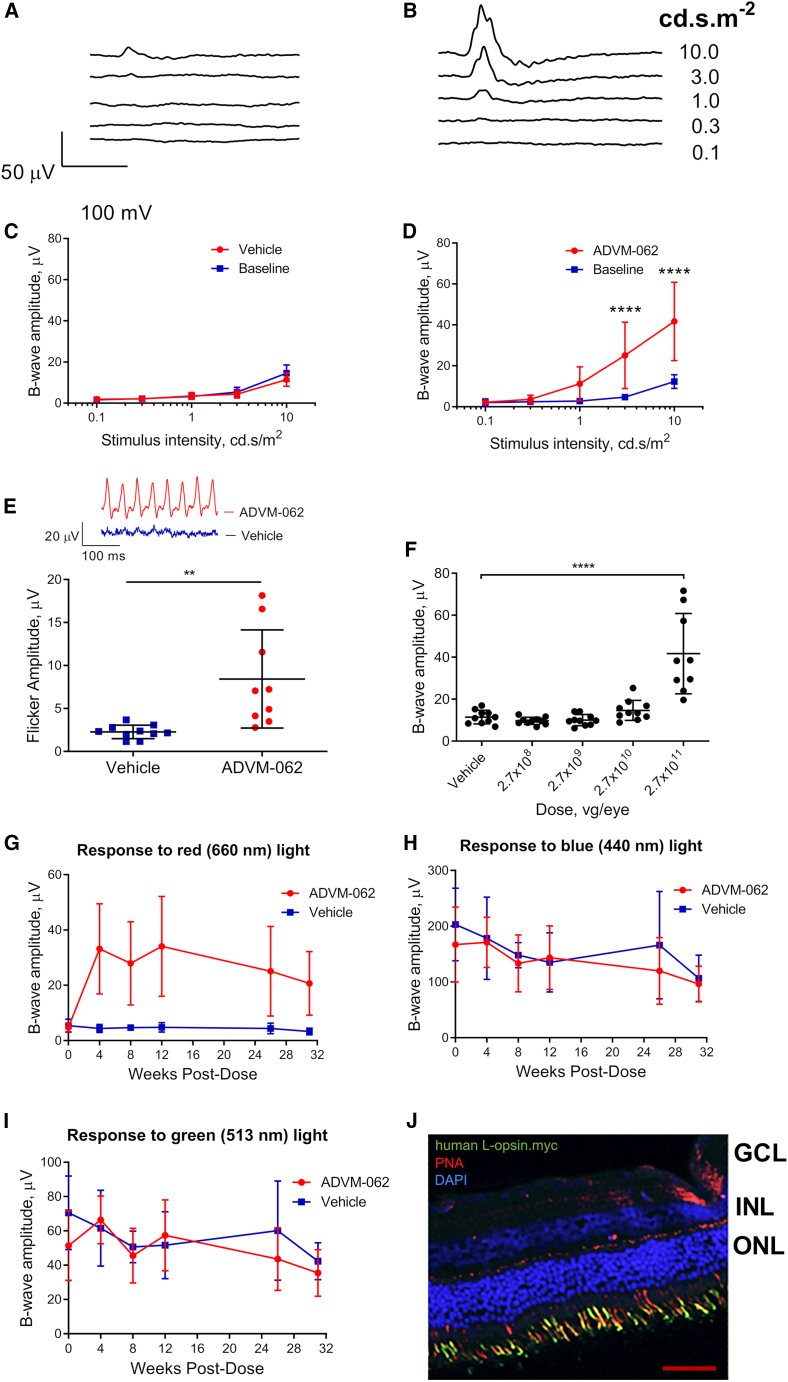
Effect of IVT dose of ADVM-062 on ERG responses to long-wavelength light in gerbils Representative ERG responses to 660 nm LED stimuli of increasing intensity in vehicle (A) and ADVM-062-administered eyes at the dose of 2.7 × 10^11^ vg/eye (B). ERG b-wave amplitude in response to increasing intensities of 660 nm light at baseline and 26 weeks post-dose in vehicle (C) and ADVM-062-administerd gerbil eyes (D) (∗∗∗∗p < 0.0001, RM two-way ANOVA with Bonferroni multiple comparisons test). ERG responses of vehicle and ADVM-062-administered gerbil eyes to 25 Hz 660 nm light flicker (E). Top: representative ERG responses of vehicle (blue trace) and ADVM-062-administered eyes (red trace). Bottom: amplitudes of 25 Hz flicker ERG responses in vehicle and ADVM-062-administered eyes (∗∗p < 0.005, unpaired t test). Dependency of the augmentation of ERG b-wave amplitude on the IVT dose of ADVM-062. ERG responses were elicited by short flashes of 660 nm light at 10 cd s m^−2^ (F). Data recorded 12 weeks post-dose are shown (∗∗∗∗p < 0.0001, ANOVA with Bonferroni multiple comparisons test). Durability of augmented ERG sensitivity to 660 nm light in the ADVM-062-administered group, measured up to 32 weeks (G). cERG responses to short- (440 nm) (H) and mid-wavelength (513 nm) (I) stimuli measured up to 32 weeks, in the same animals. Means ± SD are shown. In all ERG experiments, animal and eye numbers in vehicle control, 2.7 × 10^8^, 2.7 × 10^9^, and 2.7 × 10^10^ vg/eye groups were n = 5 animals, 10 eyes. In the 2.7 × 10^11^ vg/eye group n = 5 animals, 9 eyes (1 eye excluded due to an injection procedure-induced cataract). IVT ADVM-062.myc (5 × 10^10^ vg/eye) demonstrates cone-specific expression of human OPN1LW-myc transgene expression in the gerbil retina (J) as shown by the overlap (yellow) in myc immunostaining (green) with a pan-cone marker PNA (red). Blue, DAPI, nuclei. ONL, outer nuclear layer; INL, inner nuclear layer; GCL, ganglion cell layer. Scale bar represents 100 μm.

*In vivo* functional activity of ADVM-062 was assessed in Mongolian gerbils, a rodent model suitable for testing cone-targeted therapies. These animals have cone-rich retinas (12%–15% of photoreceptors are cones), which consist of short- (S; sensitive to ultraviolet wavelength of light) and middle (M)-wavelength-sensitive opsins but lack long-wavelength-sensitive L-opsin.^30^ The native ability of the gerbil retina to respond to red light is thus very limited, allowing for the functional evaluation of ADVM-062. Functional expression of human L-opsin was evaluated using full-field color electroretinography (cERG) with light-emitting diodes (LEDs) of different wavelengths, at different intensities and frequencies (Figure 3). A green background illumination (513 nm, 30 cd/m^2^) was used to suppress rod activity and to reduce the sensitivity of cones expressing M-opsin to long-wavelength light. Eyes administered a single IVT injection of ADVM-062 (2.7 × 10^11^ vg/eye) demonstrated significantly increased retinal sensitivity to red light (Figures 3B and 3D). Cone-isolating 25 Hz flicker cERG responses to 660 nm flashes were recorded to confirm the cone-specific nature of the ADVM-062-mediated sensitivity to red light. The significant increase in 25 Hz flicker cERG response to 660 nm light found in the ADVM-062-administered eyes was consistent with a response driven by cone cells (Figure 3E). Vehicle-injected gerbil eyes adapted to medium-wavelength light had low cERG responses to long-wavelength (660 nm) light flashes applied at intensities between 0.1 and 10 cd s/m^2^ (Figures 3A and 3C). A small increase observed at higher stimulus intensities (above 1 cd s/m^2^) was likely mediated by gerbil M-opsin. Importantly, ADVM-062 driven L-opsin expression in the gerbil retina did not adversely affect function of M- or S -cones as evidenced by unchanged cERG responses to short-wave 440 nm and mid-wave 513 nm light (Figures 3H and 3I). The effect of ADVM-062 on the retinal sensitivity to long-wavelength light peaked by 4–12 weeks, with a trend toward reduced response observed by study end (week 32) (Figure 3G), although there were not statistically significant differences between peak values and values at the end of the study as determined by one-way ANOVA with Bonferroni’s multiple comparisons test. This trend was likely related to the age-related decline in cone-based vision, similar to what has been shown in mice,^31^ since it was similar to a decline in M- and S-opsin-driven cone (Figures 3H and 3I) responses, seen in both ADVM-062- and vehicle-administered control animals. Dose-response evaluation of IVT-administered ADVM-062 in gerbils using doses of 2.7 × 10^8^ to 2.7 × 10^11^ vg/eye showed an increased ERG response over background, starting at a dose of 2.7 × 10^10^ vg/eye (17.04 ± 4.86 μV in ADVM-062-dosed versus 12.28 ± 2.91 μV in vehicle-dosed eyes; n = 10 eyes per group, mean ± SD) and reaching statistical significance at 2.7 × 10^11^ vg/eye (51.12 ± 22.07 μV, mean ± SD, p < 0.0001) in ADVM-062-treated eyes (Figure 3F). MNTC-driven cone-specific expression of human L-opsin was determined using an epitope-tagged ADVM-062 vector (ADVM-062.myc) engineered to express human L-opsin with a C-terminal myc-tag. Use of the myc-tag was necessary as there are no commercially available antibodies that differentiate between the human L-opsin transgene and either rodent M-opsin or NHP L- or M-opsins. It has been previously shown that addition of a myc-tag at the C terminus of L-opsin results in expression of functional protein.^20^ Similar to ADVM-062 (without the myc-tag), IVT ADVM-062.myc resulted in the expression of functional tagged L-opsin in the gerbil, as evidenced by the augmented ERG responses to 660 nm light stimuli (Figure S2) thus corroborating the functionality of the myc-tag human L-opsin transgene. Staining of retinal sections from ADVM-062.myc-dosed gerbil eyes showed cone-specific expression of human myc-tag L-opsin, localized to cone photoreceptor outer segments (identified by peanut agglutinin [PNA] staining, Figure 3J).

### Dose-dependent expression of human L-opsin in the NHP retina

To establish the efficacy and tolerability of IVT-delivered ADVM-062 that could translate to clinical doses, we tested different doses of the vector via IVT injection in NHPs. The detection of activity of human L-opsin in retina of NHPs with trichromatic vision is challenging due to the presence of endogenous L-opsin with a spectrum of absorbance similar to that of human L-opsin, as well as a nearly identical protein (98%) and nucleotide sequence (96%) identity between human and NHP L/M-opsins. Therefore, we utilized the ADVM-062.myc surrogate vector strategy to evaluate localization of human L-opsin and to estimate the percentage of human L-opsin-positive cones in the NHP retina.

**Figure 4:**
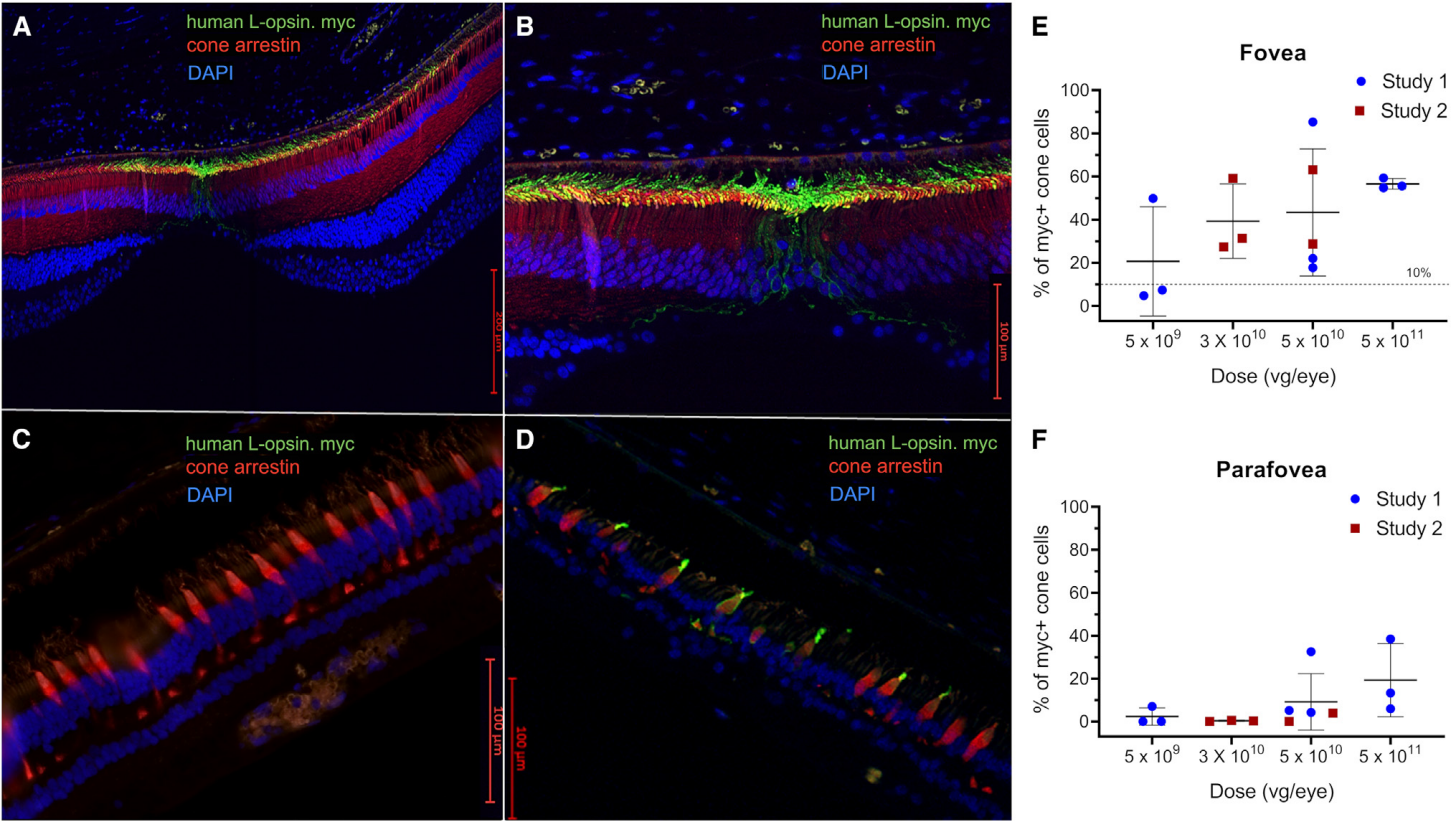
Transduction of cone photoreceptors in the NHPs dosed IVT with ADVM-062.myc The dose was delivered to the mid-vitreous (study 1) or close to the posterior pole of the eye (study 2). Localization of ADVM-062.myc-driven expression of human L-opsin.myc in cone photoreceptors of the macula (A), fovea (B), mid-periphery (C) and periphery (D) in an NHP retina (animal 1.3003, dosed at 5 × 10^10^) identified by myc-tag immunofluorescence (A–D). Blue, DAPI, nuclei; red, cone arrestin; green, human OPN1LW-myc. Scale bars represent 200 μm (A) or 100 μm (B–D). The dose-dependent frequency of L-opsin-myc-positive cones was evaluated in animals administered ADVM-062 at 5 × 10^9^ (n = 3 animals, 3 eyes), 3 × 10^10^ (n = 3 animals, 3 eyes), 5 × 10^10^ (n = 5 animals, 5 eyes), or 5 × 10^11^ (n = 3 animals, 3 eyes) vg/eye (E and F). The dose-dependent percentage of human L-opsin-positive cones in the fovea, enumerated in sections cut through the principal axis of the fovea along the fovea-optic disc axis, in the individual animals (E). Levels were quantified as the percentage of L-opsin.myc-positive cone outer segments identified by cone-arrestin counterstaining, in a 500 μm region centered over the foveal avascular zone (FAZ). The percentage of human L-opsin-positive cones in the parafovea in sections cut through the principal axis of the fovea along the fovea-optic disc axis, in the individual animals (F). Parafoveal cone cell counts were assessed in a 500 μm area selected as two 250 μm regions starting from the margins of the FAZ and extending out toward the temporal and nasal parafoveal periphery. Means ± SD are shown.

We conducted two dose-range studies using ADVM-062.myc that evaluated the dose-dependent protein localization in foveal cones and the percentage of transduced foveal cones. The enumeration of NHP cones expressing the human L-opsin served as a surrogate for evaluating the potential efficacy of ADVM-062. In study 1, ADVM-062.myc was administered at three dose levels (5 × 10^9^, 5 × 10^10^, and 5 × 10^11^ vg/eye) (Table S2). In this study, the injection needle was directed toward the mid-vitreous. Study 2 evaluated doses at 3 × 10^10^ and 5 × 10^10^ vg/eye (Table S2) using a modified IVT administration, with the injection needle directed toward the posterior pole of the eye, aiming closer to the optic disc region. The modified IVT injection was utilized to assess whether the delivery of the dose in proximity to the fovea could result in less variability across a dose group given better localized foveal cone human L-opsin levels with less parafovea transduction. Two months post-injection, immunostaining of the retina from the NHPs administered ADVM-062.myc demonstrated dose-dependent myc-tag human L-opsin levels concentrated in the fovea, which contains densely packed cones responsible for high-acuity vision (Figure 4). Representative images of foveal, mid-retinal, and peripheral (Figures 4A–4D) retinal sections from an NHP dosed with ADVM-062.myc at 5 × 10^10^ vg/eye, 8 weeks post-dose, stained with a myc-tag antibody and counterstained with the pan-cone marker cone arrestin (arrestin-C) are shown. Frequency of L-opsin-myc-positive cones was evaluated by counting myc-positive cones identified by immunofluorescence in serial histological sections cut through the fovea avascular zone (FAZ) to the periphery. Frequencies measured in the 500 μm region centered over the FAZ showed that the lowest dose of ADVM-062.myc (5 × 10^9^ vg/eye) resulted in variable transduction of foveal cones ranging from 4.8% to 49.8% in individual animals, while ADVM-062.myc at doses of 3 × 10^10^, 5 × 10^10^, or 5 × 10^11^ vg/eye resulted in transduction of foveal cones between 17.7% and 85.3% (Figure 4E). With increasing vector doses, cone transduction extended outward toward the periphery, with increasing percentage of L-opsin-myc-positive cones in the parafovea (Figure 4F) and transduction of cones observed in the periphery (Figure S3). Importantly, no expression of human L-opsin.myc was found in any retinal cells other than the cones. Both injection modalities, to mid-vitreous and in proximity to the posterior retina, resulted in similar cone transduction; however, the latter modality trended toward more consistent transduction of foveal cones in the individual animals, although the small number of the animals in the studies does not allow comparison of the efficacy of both delivery methods quantitatively. To determine whether ADVM-062 drives expression of transgenic human L-opsin in all cones or has a preference for M and L cones, a comparison of the percentage of S cones positive for human L-opsin.myc to the percentage of human L-opsin.myc-positive cells among all cone types in NHPs using the pan-cone marker arrestin-C was performed. Costaining for myc-tag and S-opsin identified S-opsin + L-opsin.myc double-positive cones, although the frequency of the S cones positive for human L-opsin was lower than the frequency of the human L-opsin-positive M and L cones normalized to their respective cone populations (Figure S4).

### Tolerability in non-human primates

NHPs administered ADVM-062.myc at 5 × 10^9^, 3 × 10^10^, and 5 × 10^10^ vg/eye across both studies demonstrated no test-article-related ocular events beyond those observed in the vehicle group (Figure S5). In eyes dosed with the highest dose of 5 × 10^11^ vg/eye of ADVM-062.myc, signs of self-limiting inflammation were observed, characterized by vitreous cell infiltrates ranging from grade 0.5+ to 2+, and a single incidence of vitreous haze in one of the eyes at day 24. The inflammation was reduced to trace levels (grade 0.5+) by the end of the study in all animals in the high-dose group. No anti-inflammatory prophylaxis or treatment was warranted at any time during the study. Based on the dose-finding studies, we evaluated the tolerability of ADVM-062 in a GLP toxicology study (Table S3). In this study, a single bilateral IVT injection of ADVM-062 was administered to 2-year-old male NHPs (n = 3 per group) at 5 × 10^10^, 1 × 10^11^, or 3 × 10^11^ vg/eye. The injection needle was directed toward the posterior pole of the eye, aiming closer to the optic disc region (using the same IVT injection modality as in the dose-range study 2 with ADVM-062.myc). Vehicle was administered via a single bilateral IVT injection to a control group (n = 2 per group). No anti-inflammatory steroids were used at any point of the study. The study duration was 14 weeks. Ophthalmic examinations (slit lamp biomicroscopy and indirect ophthalmoscopy) and tonometry were performed throughout the course of the study. ERG and optical coherence tomography were performed prior to injection and during weeks 4 and 12. On day 24, there was an early mortality for an animal administered the dose of 5 × 10^10^ vg/eye. The early mortality was unrelated to the test article and attributed to aspiration of gastric contents during recovery from sedation following a study procedure. All other animals survived to the scheduled necropsy (day 98). Overall, IVT administration of ADVM-062 was well tolerated. A single IVT injection of ADVM-062 at up to 3 × 10^11^ vg/eye produced no test-article-related changes in the following parameters: clinical observations, qualitative food consumption, body weight, intraocular pressure (IOP), ERG, electrocardiology, clinical pathology parameters (hematology, coagulation, clinical chemistry, and urinalysis), gross pathology, or organ weight. There were no ADVM-062-related changes to the retina based on evaluation of optical coherence tomography (OCT) images, including infrared plus OCT, blue reflectance, BluePeak autofluorescence, and fluorescein angiography.

**Figure 5:**
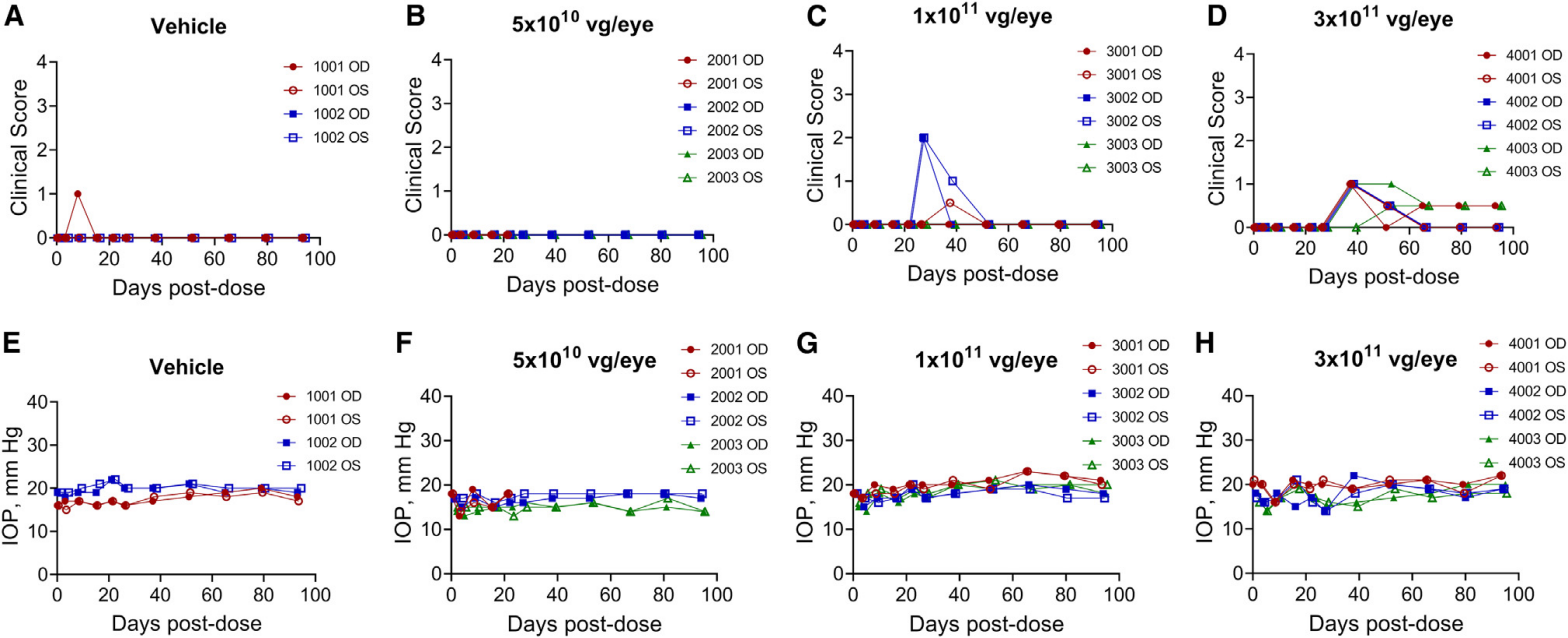
Ocular findings in the GLP toxicology study in NHPs Vehicle (A) or IVT ADVM-062 at 5 × 10^10^ (B), 1 × 10^11^ (C), or 3 × 10^11^ vg/eye (D). Vehicle group, n = 2 animals, 4 eyes; all ADVM-062 groups, n = 3 animals, 6 eyes per dose group. Clinical scores of vitreous cell infiltrates and pigment are shown (A–D). No other manifestations of inflammation, such as aqueous chamber cellular infiltrates, aqueous flare, vitreous haze, or keratic precipitate, were observed. No adverse IOP changes were observed at any time point of the study (E–H).

In-life observations were limited to dose-dependent non-adverse slight to mild ocular inflammation characterized by pigment and cells in the vitreous humor. Administration of ADVM-062 at 5 × 10^10^ vg/eye (low dose) resulted in no ophthalmic, macroscopic, or microscopic findings. The two higher doses resulted in slight to mild ocular inflammation characterized by pigment and cells in the vitreous. At the dose of 1 × 10^11^ vg/eye (mid-dose), there was a transient incidence in two of three animals of grade 2+ vitreous cells and pigment, which self-resolved during the study. The dose of 3 × 10^11^ vg/eye (high dose) resulted in trace to mild levels of pigment and cells in the vitreous (Figures 5A–5D) observed in three of three animals, which was persistent. No aqueous flare, aqueous cells, keratic precipitates, or vitreous haze was detected at any dose level. No abnormalities of the anterior segment or lens were found, and no iris changes were observed by transillumination. There were no adverse ocular tonometry findings in any treatment groups (Figures 5E–5H). There were no test-article-related histopathological findings in ocular tissues of low- or mid-dose treatment group animals. In the highest-dose group, ADVM-062-related microscopic findings were limited to minimal mononuclear infiltrates within the superficial optic disc (observed in both right eyes selected for histopathological analysis). Microscopically, lymphocytes, macrophages, and occasional plasma cells were observed perivascularly and extending into the adjacent neuropil of the optic disc. These findings were considered non-adverse based on their minor severity, in agreement with the absence of abnormal findings in ocular physiology and function in the animals (Figure S6). Other microscopic findings observed were considered incidental, of the nature commonly observed in NHPs.^32^^,^^33^^,^^34^ They were of similar incidence and severity in control and dosed animals and thus considered unrelated to the administration of ADVM-062. Based on the results of the GLP toxicology study, the no observed adverse effect level (NOAEL) was established at 3 × 10^11^ vg/eye, the highest dose tested in the study.

## Discussion

A gene therapy approach that can deliver sustained levels of a functional copy of L-opsin is anticipated to improve BCM symptoms in patients affected by this debilitating, inherited retinal cone dystrophy. Subretinal delivery of AAV2 gene therapy vectors has been commonly considered as a way to efficiently transduce the distal retina and retinal pigment epithelium.^35^^,^^36^^,^^37^ However, for BCM patients, who have varying degrees of macular thinning and still maintain some level of visual acuity, the risk of foveal damage and potential subsequent complete loss of central vision that is associated with subretinal surgery is undesirable.^12^^,^^13^^,^^22^ New variants of AAV, such as AAV.7m8, have been developed to efficiently transduce the fovea following a single IVT injection.^23^ These IVT AAV vectors may allow the development of gene therapy products that are better suited in terms of risk:benefit ratio for patients with retinal cone diseases. ADVM-062 is a gene therapy product specifically designed to deliver a functional copy of the human L-opsin gene via the AAV.7m8 vector to the foveal cones of patients suffering from BCM via a single IVT injection. Tropism studies using the AAV.7m8 vector delivered via a single IVT injection to the NHP eye demonstrated strong transduction of the retinal areas known to have a thin inner limiting membrane (such as the fovea and peripheral retina, including foveal cones).^23^^,^^24^^,^^25^^,^^38^ Expression of functional human L-opsin using ADVM-062 in the foveal region is expected to improve central vision and visual acuity in BCM patients, while expression of human L-opsin in the cones of peripheral retina could provide some additional benefit, given that peripheral cones appear to play a role in the perception and processing of color and motion information out to the far periphery of the visual field.^39^ From the tolerability perspective, ADVM-062 was designed to drive the expression of human L-opsin exclusively in the cone photoreceptors, sparing other retinal neurons. Using AAV.7m8-MNTC-GFP delivered IVT to the NHP eye, we demonstrated cone-specific expression of human L-opsin mediated by the MNTC regulatory elements.^26^ Given the trichromatic nature of NHPs, the pharmacological activity of ADVM-062 was evaluated in dichromatic Mongolian gerbils, a rodent species with a cone-rich retina that express only short- (UV) and middle-wavelength-sensitive opsins.^40^^,^^41^ The gerbil’s limited innate ability to respond to long-wavelength (red) light was significantly and durably augmented by IVT-injected ADVM-062. The effect of ADVM-062 in gerbils showed a steep dose response and reached statistical significance at 2.7 × 10^11^ vg/eye. The steep dose response observed in gerbils could result from the different ocular anatomy of small rodents compared with NHPs and humans and the possible vector transit through the gerbil inner limiting membrane and several retinal cell layers in the fovea-less retina to reach the cone photoreceptors. In gerbils administered ADVM-062, ERG responses to 660 nm light using 25 Hz flicker confirmed the cone-specific nature of long-wave sensitization. Further, immunofluorescence studies conducted on retinas of animals administered ADVM-062.myc confirmed that human L-opsin expression was localized to outer segments of cone photoreceptor cells. These results provide strong evidence that IVT-administered ADVM-062 is effective in generating functional human L-opsin in cone photoreceptors. Given the significant differences in the ocular anatomy between the primate and the rodent retina and the substantial differences in the immune system/inflammation response of these species, human clinical dose extrapolation was evaluated in NHPs.^28^^,^^42^ To establish first-in-human clinical doses and assess tolerability, ADVM-062 was evaluated in NHPs, a species whose overall retinal structure, including the presence of a fovea, closely resembles that of humans.^28^ Measuring expression, subcellular localization, and pharmacological activity of human L-opsin on the background of the trichromatic NHP retina is challenging, due to an inability to differentiate and quantitate human L-opsin from the endogenous, functionally identical NHP opsin that shares 98% sequence identity with human protein. To overcome these constraints we generated the AAV.7m8 vector, which carries the expression cassette for human L-opsin with the same regulatory elements as ADVM-062 but with an addition of a C-terminal myc-tag, to determine localization and to quantify percentage of foveal cone transduction as a proxy for the pharmacologically active levels of ADVM-062. We evaluated the percentage of foveal cones that produce human L-opsin in the NHP retina as a relevant metric for ADVM-062 activity to guide the selection of first-in-human doses. The use of ADVM-062.myc confirmed cone-specific human L-opsin levels in the NHP retina at all doses tested. The exploration of the doses yielding efficient cone transduction revealed that at a low dose of vector (5 × 10^9^ vg/eye), variable transduction was achieved, from very few transgene-positive cones to approximately half of foveal cones transduced in a section cut through the fovea center (Figure 4). Hypothetically, the observed variability at low doses could result from a variety of mechanisms, such as neutralizing activity toward the AAV vector, potential heterogeneity of vitreous body density affecting vector dispersal, or variability in the inner limiting membrane (ILM) thickness of individual animals. The doses of 3 × 10^10^ vg/eye and above resulted in a potentially clinically meaningful transduction. At these doses, the densities of transduced cones at the lowest level exceeded the number of residual cones in patients with Stargardt’s disease, an autosomal recessive macular dystrophy, sufficient to maintain useful visual acuity^43^^,^^44^^,^^45^^,^^46^ and were up to the density levels suggested to be sufficient for high visual acuity.^45^^,^^46^^,^^47^

However, outstanding questions remain on the path to the clinic around the percentage of foveal cone transduction required for visual function gains and the expression level of exogenous L-opsin required, together with whether the visual system of BCM patients will be able to process new signals from congenitally inactive cones. The percentage of foveal cone transduction required for sufficient visual function can potentially be inferred from clinical studies in patients. There are a number of potential studies to draw information from, including the number of surviving cones in patients with retinal degenerative diseases, studies in individuals with X-linked dichromacy who have various numbers of functional cones, and finally, neurophysiological studies in healthy individuals. For example, in Stargardt’s disease, it was estimated that significant changes in visual acuity result only following the loss of approximately 90% of the cone photoreceptors.^43^^,^^44^ Adaptive optics SLO (ASLO) studies that measured the relationships between cone density and visual acuity in patients with several different retinal degenerative diseases found that visual acuity in patients was affected by cone loss when cone densities were 52%–62% or 40%–50% below normal average, respectively.^45^^,^^46^ In the study by Seiple et al.,^47^ photoreceptor losses were modeled in healthy subjects by blanking randomly selected pixels. The study suggested that patients with a cone loss of 50% or more would be able to maintain 20/20 acuity, and 96% random loss of photoreceptors would result in visual acuity at 20/60. In addition, information about the cone density sufficient to support foveal vision can be obtained from studies of humans with X-linked red-green color vision deficit. It has been shown that the relative number of L and M cones (L:M ratio) varies greatly across individuals,^48^^,^^49^^,^^50^ and inactivation of one opsin gene (L or M) results in a variable degree of disruption of the foveal cone mosaic, likely due to variability in the L:M cone ratio. Studies of the relationship of foveal cone density with visual acuity in human dichromats demonstrated that differences in cone density were not apparent functionally.^50^ Interestingly, a patient in that study with very low cone peak density of 18,927 cones/mm^2^ had normal visual acuity, comparable to another subject with normal trichromatic vision, and a density of 195,030 cones/mm^2^. This value was also significantly lower than the average peak cone density in the foveal area of 164,000 ± 24,000 cones/mm^2^,^51^ as determined by ASLO. These data indicate that the restoration of function of ∼10%–17% of foveal cones may be sufficient to achieve clinical benefit in BCM patients. Ultimately, only clinical studies in human subjects will illuminate the percentage of foveal cones required for gains in visual function.

The level of expression of transgenic L-opsin is another parameter that can determine the efficacy of the ADVM-062 dose. The minimal level of exogenous cone L-opsin protein production per cone cell required to generate responses to light is not known, and the close similarity of human L-opsin to NHP L- and M-opsins did not allow us to compare the levels of transgenic L-opsin with the levels of endogenous visual opsins per cone cell. According to the studies in mouse models, 10%–20% of normal expression of visual pigment in cone or rod photoreceptors may be sufficient to maintain the photoreceptor structure and function.^52^^,^^53^^,^^54^ Potentially, even expression of visual opsin below normal levels in cones may be beneficial in human patients. The study of BCM patients with various degrees of severity of disease suggested that low residual levels of visual opsin expression can alleviate some of the disabling symptoms of BCM.^13^ As previously mentioned, it is not yet clear whether the visual system of patients will be able to process new signals from congenitally inactive cones such as those associated with deletion and mutations associated with BCM. The evaluation of L-opsin.myc expression in L/M and S cones indicated that S cones were somewhat permissive for MNTC regulatory cassette activity. Similarly, promoters based on the human L-opsin promoter containing LCR with progressive truncations were shown to be active not only in L/M cones but also in S cones.^55^ While the mechanism of the promiscuous activity of LCR and L- or M-opsin promoter sequences in L/M and S cones is unclear, it is plausible that the synthetic expression cassettes based on these promoters have impaired interactions with putative S-cone-specific suppressors of L/M-opsin expression.^56^ BCM patients could potentially benefit from the expression of L-opsin in S cones, the only functional cones in the patients’ retina. BCM patients’ vision strongly relies on the S cones, which have increased density at the locus of fixation, which is slightly offset from the fovea in BCM patients,^57^ and/or elevated fovea S-cone counts observed in the BCM patients’ fovea.^13^ In theory, the expansion of the spectral sensitivity of functional S cones in BCM retina toward long-wavelength light could result in improved visual function in BCM patients. In some mammals, such as mice, most of the cones coexpress M- and UV/S-opsins, to broaden the spectral range to which a cone cell is sensitive.^58^

In conclusion, the totality of data in support of ADVM-062 as a single-administration therapy for BCM is supported by the functionality of ADVM-062 in a dichromatic small-rodent model together with NHP data showing potentially clinically meaningful levels of foveal cone transduction at well-tolerated doses in NHPs. The GLP toxicology study demonstrated that ADVM-062 was well tolerated, with the NOAEL established at 3 × 10^11^ vg/eye (human equivalent dose (HED) 6 × 10^11^ vg/eye). These data indicate that ADVM-062 may display a favorable risk:benefit ratio and support the clinical development of ADVM-062 as an IVT gene therapy for BCM patients.

## Materials and methods

### Vectors

The studies reported here used three different recombinant single-stranded AAV constructs, flanked by AAV2 ITRs packaged in the AAV.7m8 capsid variant: (1) ADVM-062, (2) ADVM-062.myc, and (3) AAV.7m8-MNTC-GFP, which expresses the EGFP reporter gene under the control of the MNTC promoter. The synthetic MNTC cassette includes an LCR enhancer sequence from the human L- and M-opsin genomic locus and a truncated promoter sequence from the human M-opsin gene, comprising about 140 nucleotides upstream of the transcriptional start site. In addition, the cassette includes a 5′ UTR based on the M-opsin 5′ UTR but modified to have minimal secondary structure and to include additional sequence at its 3′ end, into which an intron was inserted. The human *OPN1LW* (L-opsin) cDNA sequence is followed by a 3′ UTR that corresponds to the downstream genomic region of human M-opsin. Also included in the pMNTC polynucleotide cassette is a strong Kozak sequence and an SV40 polyadenylation sequence.^27^

To enable detection of AAV.7m8-MNTC-driven expression of the transgene *in vivo* using SLO, and *ex vivo*, we utilized AAV.7m8-MNTC-GFP. To enable detection and localization of human L-opsin in the presence of endogenous opsins and to quantify the efficacy of transduction based on percentage of human L-opsin-positive cones, we engineered ADVM-062.myc to express human L-opsin with a C-terminal myc-tag. With the exception of the myc-tag inserted in frame into the *OPN1LW* coding sequence, ADVM-062.myc was identical to ADVM-062. To ensure comparability of results acquired from the animals administered either vector, the ADVM-062 and ADVM-062.myc vectors were produced and characterized using similar processes and protocols.

Both ADVM-062 and ADVM-062.myc were manufactured in the baculovirus expression vector system (BEVS)^59^ in an Sf9 working cell bank (WCB). In this system, two baculoviruses are used, one encoding the AAV2 Rep and AAV.7m8 Cap proteins and another encoding the rAAV genome carrying the human L-opsin cDNA and MNTC expression cassette. AAV.7m8-MNTC-GFP was produced using the triple-transfection method in HEK293 cells and purified by iodixanol gradient ultracentrifugation.^60^ rAAV vectors were titered by quantitative PCR (qPCR) using TaqMan probes (Thermo Fischer Scientific).

### Animals and study design

#### Characterization of the cone specificity and efficiency of the AAV.7m8 capsid combined with MNTC regulatory elements using the surrogate vector AAV.7m8-MNTC-GFP in African green monkeys (*Chlorocebus sabaeus*)

The study with AAV.7m8-MNTC-GFP was conducted in adult (4–10 years of age) African green monkeys of both sexes (n = 2 total). All animals were used in accordance with the ARVO Statement for the Use of Animals in Ophthalmic and Vision Research. The use of bilateral treatments and all procedural aspects of the animal studies were approved by the Institutional Animal Care and Use Committee (IACUC) overseeing animal welfare at the primate facility (St. Kitts Biomedical Research Foundation, St. Kitts, West Indies) with which Virscio (New Haven, CT) maintains a facility use agreement. NHPs were anesthetized with intramuscular injection of 8.0 mg/kg ketamine/1.6 mg/kg xylazine and mydriasis was achieved with topical 10% phenylephrine. An eye speculum was placed in the eye to facilitate injections. For IVT injections (n = 4 eyes/condition), 50 μL of AAV.7m8-MNTC-GFP vector was delivered to yield a final dose of 5 × 10^11^ vg/eye. IVT injections into the central vitreous were administered using a 31G 3/8 inch needle inserted inferotemporally at the level of the ora serrata ∼2.5 mm posterior to the limbus. Central vitreous placement was confirmed by direct observation of the needle tip at the time of the injection. Following IVT injections, a topical triple-antibiotic ointment was administered. Retinal examination and fundus color imaging were performed by using a Topcon TRC-50EX retinal camera with Canon 6D digital imaging hardware and New Vision Fundus Image Analysis System software and Spectralis OCT Plus. Characterization of cone cell specificity of the GFP transgene was achieved using immunofluorescence staining of retina cryosections, as described under “immunohistochemistry.”

#### Cynomolgus monkeys (*M. fascicularis*)

All animals were used in accordance with the ARVO Statement for the Use of Animals in Ophthalmic and Vision Research. The GLP-compliant toxicology study and Study 1 were reviewed and approved by the Charles River Laboratories Reno IACUC, OLAW Assurance no. D16-00594. Study 2 was reviewed and approved by the Biomere IACUC, OLAW Assurance no. D16-00632. The care and use of animals was conducted according to the guidelines of the US National Research Council on Animal Care.

All animals were in the normal range at baseline ophthalmic screening, including tonometry, slit-lamp biomicroscopy, fundoscopy, fluorescein angiography (FA), and OCT.

*ADVM-062.myc expression and tolerability studies.* These were conducted in 2.5- to 2.8-year-old male NHPs. All animals recruited in the study were pretested for the neutralizing antibody (Nab) using the IC_50_ method.^61^^,^^62^^,^^63^ In both studies animals were randomized into four treatment groups, three per group in study 1 and two per group in study 2, by weight and Nab titer (Table S2). In study 1, group 1 was administered IVT-injected vehicle. Groups 2, 3, and 4 were administered ADVM-062.myc injected IVT in 50 μL at 5 × 10^9^, 5 × 10^10^, and 5 × 10^11^ vg/eye, respectively. Animals characterized as Nab-positive but with low IC_50_ values were recruited to this study and distributed, together with Nab-negative animals, among the ADVM-062-treatment groups. All animals were evaluated for treatment tolerability. In ADVM-062.myc-administered groups 2, 3, and 4, the right eyes were fixed and processed for detection of human L-opsin.myc by immunofluorescence. Cornea, lens, and iris were removed, and eyecups were fixed in 10% neutral buffered formalin for 24 h followed by processing into paraffin blocks. Study 2 had expanded testing with doses of 3 × 10^10^ and 5 × 10^10^ vg/eye; all animals had negative or low Nab activity. Animals were randomized into two treatment groups. Group 1 (three animals in each) was administered ADVM-062.myc at 3 × 10^10^ vg/eye, and group 2 (two animals) was administered ADVM-062 or ADVM-062.myc injected in 50 μL at 5 × 10^10^ vg/eye to the left and right eye, respectively (Table S2). Left eyes from groups 1 and 2 (ADVM-062.myc-dosed) were analyzed by immunofluorescence for transgenic human L-opsin.myc localization and percentage of the transgene product-positive cones in fovea. The eyecups were prepared and processed as in the first study.

An additional study was conducted at Biomere in male NHPs to assess the ADVM-062-driven intraocular expression of human L-opsin transgenic mRNA, as described under “*in situ* hybridization.”

*GLP-compliant toxicology study.* This study recruited 11 1.8- to 2.4-year-old NHP males. All animals recruited into the study were screened for the presence of Nab using the cut-point method.^63^^,^^64^ The assay was conducted at BioAgilytix Labs (Durham, NC). Only Nab-negative animals were assigned to the ADVM-062 treatment groups in this study. Animals were randomized into four treatment groups (Table S3). Group 1 (n = 2) was administered IVT-injected vehicle to both eyes. Groups 2, 3, and 4 (n = 3 animals in each) were administered ADVM-062 injected IVT into both eyes at 5 × 10^10^, 1 × 10^11^, and 3 × 10^11^ vg/eye, respectively. The injection needle was directed toward the posterior pole of the eye, aiming closer to the optic disc region. The animals were maintained in the supine position for at least 10 min following IVT injection of the test material. All animals were evaluated for treatment tolerability. At termination, one right eye from group 1 (vehicle control) and two right eyes from each ADVM-062-treatment subgroup were fixed in Davidson’s, processed, and analyzed for histopathology. Other ocular tissues were retained for bioanalytical purposes.

#### Animal care and handling

Animals were anesthetized for all procedures and ophthalmic evaluations using intramuscular ketamine/dexdomitor. General well-being was assessed before, during, and after sedation as well as twice daily on non-procedure days. The daily consumption of food biscuits was monitored. Body weight was measured weekly or at the time of ophthalmic examinations.

#### Test article delivery

In the first study that evaluated dose-dependent transduction of cone photoreceptors, IVT doses were administered under local anesthesia (0.5% proparacaine) using a 31G 5/16 inch needle (Ulticare Vet RX U-100, no. 09436) 2 mm posterior to the limbus in the inferior temporal quadrant, targeting the central vitreous body. Injections were followed by topical administration of 0.3% ciprofloxacin ophthalmic solution and 1% atropine ophthalmic ointment. In the second study, as well as in the GLP toxicology study that assessed tolerability of ADVM-062, the dose was delivered into the posterior vitreous. The needle of the dose syringe was inserted through the superior temporal sclera and pars plana approximately 4 mm posterior to the limbus. The needle was directed posterior to the lens and advanced posteriorly toward the optic disc, and the test article was slowly injected.

#### In-life assessments

Slit-lamp biomicroscopy was used to examine the anterior segment, lens, and anterior vitreous body. Indirect ophthalmoscopy was used to examine the vitreous, fundus, and optic disc. For ERGs, animals were anesthetized, and pupils were dilated with 1% tropicamide. Animals were dark-adapted for at least 1 h before the scotopic tests and light-adapted for at least 10 min before the photopic tests. The animals were fasted at least 2 h before ERG procedures. OCT was acquired using the Heidelberg Spectralis. Scan acquisition was as follows: infrared plus OCT (IR + OCT), blue reflectance (BR), and BluePeak autofluorescence (BAF). FA was performed following intravenous (i.v.) injection of 10% fluorescein sodium (0.1 mL/kg). An SLO image was generated during the IR scan to capture representative images of each eye. At completion of the in-life part of the study, animals were euthanized by i.v. injection of a commercially available veterinary euthanasia solution (identified in the study records), followed by exsanguination. In the dose-range studies with ADVM-062.myc, ocular tissues were collected for analysis of levels of cone transduction human L-opsin expression and localization by myc-tag immunofluorescence according to the assignments made at the initiation of the study (Table S2) or, in case of the GLP toxicology study, for histopathology or biodistribution.

#### *In situ* hybridization

Specificity of expression of the MNTC-driven human L-opsin transgene from ADVM-062 was confirmed by detection of L-opsin transgene mRNA in retinal tissue of one NHP male dosed with ADVM-062 at 2 × 10^12^ vg/eye. The eye globe from the treatment-naive NHP was used as control. The animals were sacrificed 2 months post-dose. Whole globes were fixed in 10% neutral buffered formalin upon enucleation for 24 h with post-fixation in 70% ethanol, followed by processing into paraffin blocks within 72 h. Transverse sections through the optic disc and central cornea were collected at 5 μm thickness and mounted on charged slides. Slides were processed at Advanced Cell Diagnostics (ACD). To unmask ADVM-062-produced human L-opsin, the DNA signal was eliminated by DNase I pretreatment (Sigma, no. 4716728001). DNase I (10 U/μL) at 1:50 dilution in 25 mM Tris-HCl, 50% glycerol (v/v) (pH 7.6), buffer was used after optimization. ISH was performed using the BaseScope LS Red Reagent Kit (ACD, no. 323600). Custom pretreatment conditions included target retrieval of 15 min at 95 °C–100 °C, RNAscope Protease Plus (ACD, no. 322380) for 15 min at room temperature (RT), and custom DNase I treatment at a 1:50 dilution. Counterstaining with Gill’s hematoxylin for 2 min at RT was performed after probe, amplification, and chromogen steps in the LS kit assay. ISH labeling of ADVM-062 vector genome DNA and human L-opsin mRNA was performed using 3-ZZ paired probes (sense and antisense) targeted to a part of the SV40 poly(A) signal (Table S1). This non-conventional target region was selected to provide the unique hybridization tag to enable the detection of vector genome and transgenic human L-opsin mRNA in the NHP retina sections, in the presence of the NHP L-opsin nucleic acids that have very high sequence identity with transgenic human L-opsin. The 3-ZZ-probe pair design was verified to detect vector genomic DNA and mature transgenic RNA. Bright-field images were acquired with a Leica Versa 6 automated scanning microscope equipped with a 40× objective lens. Qualitative evaluation of ADVM-062 DNA and mRNA ocular biodistribution was performed using Leica Image Scope software.

#### Mongolian gerbils (*Meriones unguiculatus*)

All animals were treated in accordance with the ARVO Statement for the Use of Animals in Ophthalmic and Vision Research, and all studies were approved by the IACUC at Life Source Biomedical Services (NASA AMES Center, Mountain View, CA). Ten-week-old female Mongolian gerbils were obtained from Charles River Laboratories (Wilmington, MA). Baseline screening by fundus imaging ensured that all animals had normal ocular health. For IVT injections, the animals were anesthetized with inhaled isoflurane and 90.7 mg/kg ketamine and 2.7 mg/kg xylazine administered via an intramuscular injection. Eyes were topically treated with 0.1% dexamethasone and pupils were dilated with 1% atropine. Injection was performed using a beveled 36G needle mounted on a 100 μL Hamilton syringe connected to a microinjection pump (UMP-3 UltraMicroPump, World Precision Instruments, Sarasota, FL) to deliver 6 μL of test solution into the vitreous cavity. The needle tip was allowed to remain in the eye for 30 s to ensure complete test article dispensing. After injection, eyes were treated with topical bacitracin/atropine/0.1% dexamethasone ointments. In the vector-treatment groups, animals were injected bilaterally with ADVM-062 at doses of 2.7 × 10^8^ to 2.7 × 10^11^ vg/eye, or with ADVM-062.myc at 2.7 × 10^11^ vg/eye. In the vehicle control group, animals were administered injections of 6 μL formulation buffer (180 mM NaCl, 10 mM phosphate, 0.001% Pluronic F-68 [pH 7.3]) to both eyes. Eye examinations and fundus photography were performed by using a slit lamp (Topcon) and Micron IV fundus microscope (Phoenix).

#### ERG

Gerbils were anesthetized with inhaled isoflurane and 90.7 mg/kg ketamine and 2.7 mg/kg xylazine administered via intramuscular injection. Pupils were dilated with topical atropine ophthalmic solution (1%), and one drop of GenTeal was applied to the cornea. A subdermal reference electrode was placed in the nasal septum and a ground electrode was placed subcutaneously above the base of the tail. The animals were positioned on a temperature-controlled platform (Espion Electrophysiology System, Diagnosys, Lowell, MA) with the head covered with the ColorDome E3 stimulator, customized by the manufacturer to deliver long-wavelength LED stimuli with a peak at 660 nm. The animals were adapted to ambient room light for at least 1 h. Prior to ERG, the animals were exposed for 5 min to a 513 nm full-field background at 30 cd/m^2^. All ERG procedures were conducted on the same background. Human L-opsin activity was tested using stimulation with 660 nM LED flashes at varying intensities delivered at 1 Hz. Flicker ERG responses to 660 nm stimuli at 10 cd s/m^2^ were recorded at 10 and 25 Hz. Responses to short (440 nm peak) and medium (513 nm peak) LED light flashes at 0.1 or 0.5 cd s/m^2^, respectively, were recorded to evaluate whether the expression of human L-opsin from ADVM-062 affected the normal function of cones in gerbil retina.

#### Immunohistochemistry

For the preparation of cryosections of ocular tissues of NHPs administered AAV.7m8-MNTC-GFP IVT, the cornea was removed, and remaining eyecups were fixed in 4% PFA for 24 h followed by post-fixation storage in 30% sucrose with 0.01% sodium azide. Eyecups were dissected into four geographical quadrants (superior, inferior, nasal, and temporal). Each quadrant was embedded in OCT medium and frozen in 2-methylbutane over dry ice. Samples were sectioned at a thickness of 7 μm. Localization studies of L-opsin.myc expressed from ADVM-062.myc were performed using formalin-fixed paraffin-embedded (FFPE) tissue samples. Gerbil whole eyes and NHP eyecups were fixed in 10% neutral buffered formalin (NBF) for 24 h upon enucleation. Tissues were prepared for paraffin embedding using vacuum infiltration processing and placed into blocks. Mid-sagittal sections through the visual streak of gerbil eyes were collected at 5 μm thickness and mounted on charged slides. During NHP eye processing, transverse sections through the optic disc and central cornea were collected at 5 μm thickness and mounted on charged slides. Slides were deparaffinized and rehydrated prior to antigen retrieval in citrate buffer. Immunofluorescent labeling was performed using a Dako LV-1 autostainer following tissue permeabilization and serum protein blocking. Antibodies and PNA used for detection of retinal markers are listed in Table S4. Images were acquired with a Hamamatsu ORCA-Flash4.0 camera on a Zeiss Axio Observer.Z1 inverted microscope equipped with a 20× objective lens. Fluorescence images employed the additional use of the Zeiss Apotome optical sectioning system using structured illumination. Images were processed and analyzed using the Zeiss Zen Blue imaging and analysis software.

#### Quantitation of transduction efficacy of fovea cones

Five serial slides with NHP eye sections were selected starting from the central fovea toward the superior parafovea at 30 (first dose-range study) or 25 μm increments (second dose-range study). After antigen retrieval, sections were immunostained with antibodies against c-myc (ab172, Abcam) and the cone-specific marker cone arrestin (AB15282, Millipore Sigma) (Table S4). NHP foveal cone cell quantifications were performed on stitched 20× enhanced resolution images. The foveal area was subdivided into two regions, the fovea and the parafovea. A 500 μm region centered over the FAZ was selected to represent foveal cone cell counts. Parafoveal cone cell counts were assessed by selecting a 550 μm region starting from the margins of the FAZ and extending out toward the temporal and nasal parafoveal periphery. Nuclei were identified using DAPI. Total cone number of human L-opsin-myc-positive cone outer segments were counted and normalized to total cone number, estimated as the number of nuclei in the outer nuclear layer of the fovea known to consist entirely of cones based on cone arrestin staining.^65^ Expression of human L-opsin-myc in S cones was enumerated as relative number of S-opsin-positive cones costained with myc-specific antibody (Table S4). Cell counting was performed by a treatment-masked investigator and replicated by a second treatment-masked investigator.

### Statistical analysis

The effect of treatment on the ERG b-wave amplitudes in response to 600 nm stimuli provided with different intensities was evaluated using repeated measures (RM) ANOVA with Bonferroni multiple comparisons test. The amplitude of response to 660 nm stimuli in animals that were administered different doses of ADVM-062 was compared with that in vehicle-administered animals and was evaluated using ordinary one-way ANOVA with Bonferroni multiple comparisons test. Amplitudes of the fundamental component of flicker ERG responses at 25 Hz in vehicle and ADVM-062-administered eyes were measured using fast Fourier transformation (FFT) and compared using an unpaired t test.

## Data and materials availability

All data generated and reported in this study are included in this published article and its supplementary information files. There are restrictions on reagent availability such as vectors generated in this study due to the limited stock, potential clinical needs, and agency requirements. The data on the ADVM-062 structure are available from the patent WO2022/204594 A1.

## Supplemental information

Document S1–S6 and Tables S1–S4

Document S2. Article plus supplemental information
