# Supplementary material for: Preclinical evaluation of ADVM-062, a novel intravitreal gene therapy vector for the treatment of blue cone monochromacy
Source: Mol Ther. 2023 Mar 16;31(7):2014–27. doi: 10.1016/j.ymthe.2023.03.011 (PMC10362383; doi:10.1016/j.ymthe.2023.03.011)
Supplement: Document S1–S6 and Tables S1–S4 [file mmc1.pdf]

YMTHE, Volume *31*

Supplemental Information

Preclinical evaluation of ADVM-062, a novel intravitreal gene therapy vector for the treatment of blue cone monochromacy

Kelly Hanna, Julio Nieves, Christine Dowd, Kristina Oresic Bender, Pallavi Sharma, Baljit Singh, Mark Renz, James N. Ver Hoeve, Diana Cepeda, Claire M. Gelfman, Brigit E. Riley, and Ruslan N. Grishanin

Supplemental Data.

Supplemental Figures.

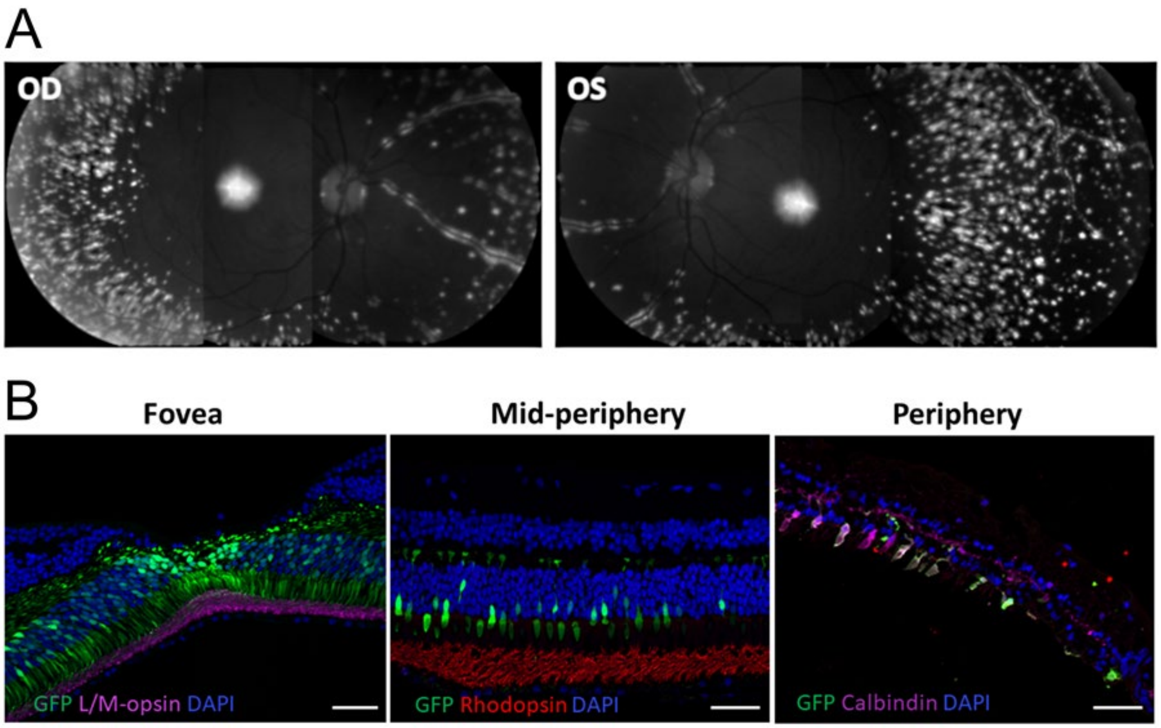

Figure S1. The MNTC regulatory cassette drives pronounced and abundant expression in the NHP cone photoreceptors. Composite fundus autofluorescence images acquired by SLO shows GFP expression in retinas of both eyes of the animal dosed with 5×10^{11} vg/eye of AAV.7m8-MNTC-GFP at 12 weeks post-IVT injection (**A**). AAV.7m8-MNTC-GFP results in cone-specific transgene expression in the NHP retina (Animal K271) in the fovea (left), mid-periphery (middle) and periphery (right) following IVT administration as shown by the overlap in GFP (green) with L/M-opsin and calbindin (marker of peripheral and perifoveal cones, magenta), Week 19 post-dose (**B**). Scale bars represent 100µm.

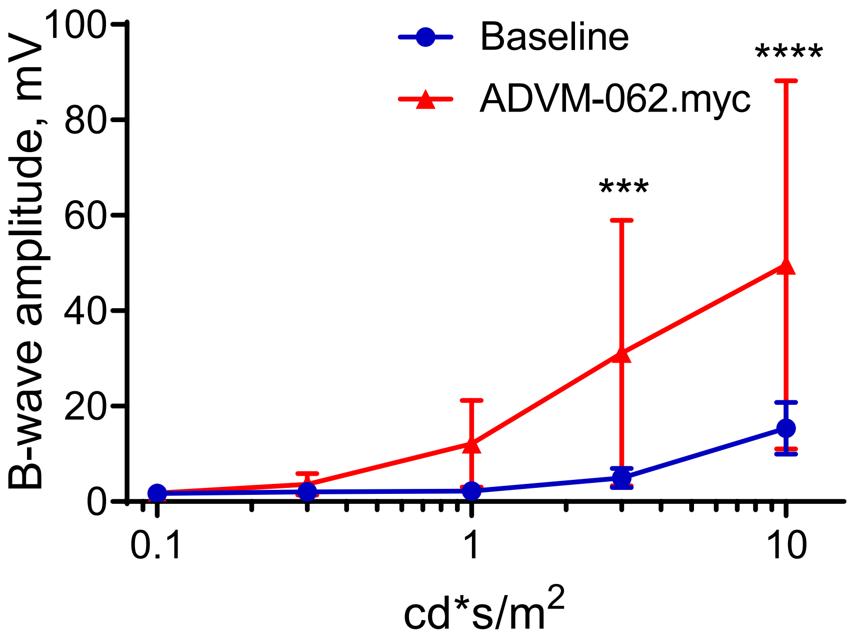

Figure S2. ADVM-062.myc sensitizes ERG responses in gerbil retina to 660 nm light stimuli, on rod and M-opsin suppressing background (***: P<0.001, ****: P<0.0001 RM 2 way ANOVA with Bonferroni multiple comparisons test). n=16 eyes, 6 animals per treatment group. Means ± SD shown.

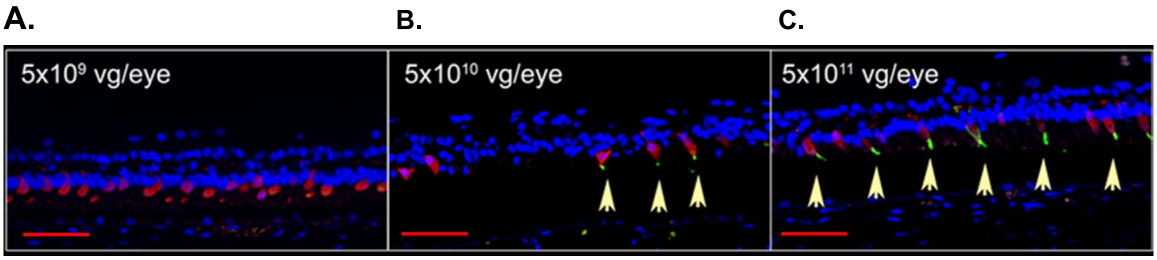

Figure S3. Dose-dependent expression of human L-opsin.myc in peripheral cones. ADVM-062.myc was IVT dosed at 5×10^9 vg/eye *(Animal 1.2001)* (**A**), 5×10^{10} vg/eye *(Animal 1.3002)* (**B**) and 5×10^{11} vg/eye *(Animal 1.4003)* **(C)**. *Blue:* DAPI, nuclei. *Red:* cone arrestin, *Green*: hOPN1LW.myc. *Arrowheads:* human L-opsin.myc-positive cones in peripheral retinas. Scale bars represent 100µm.

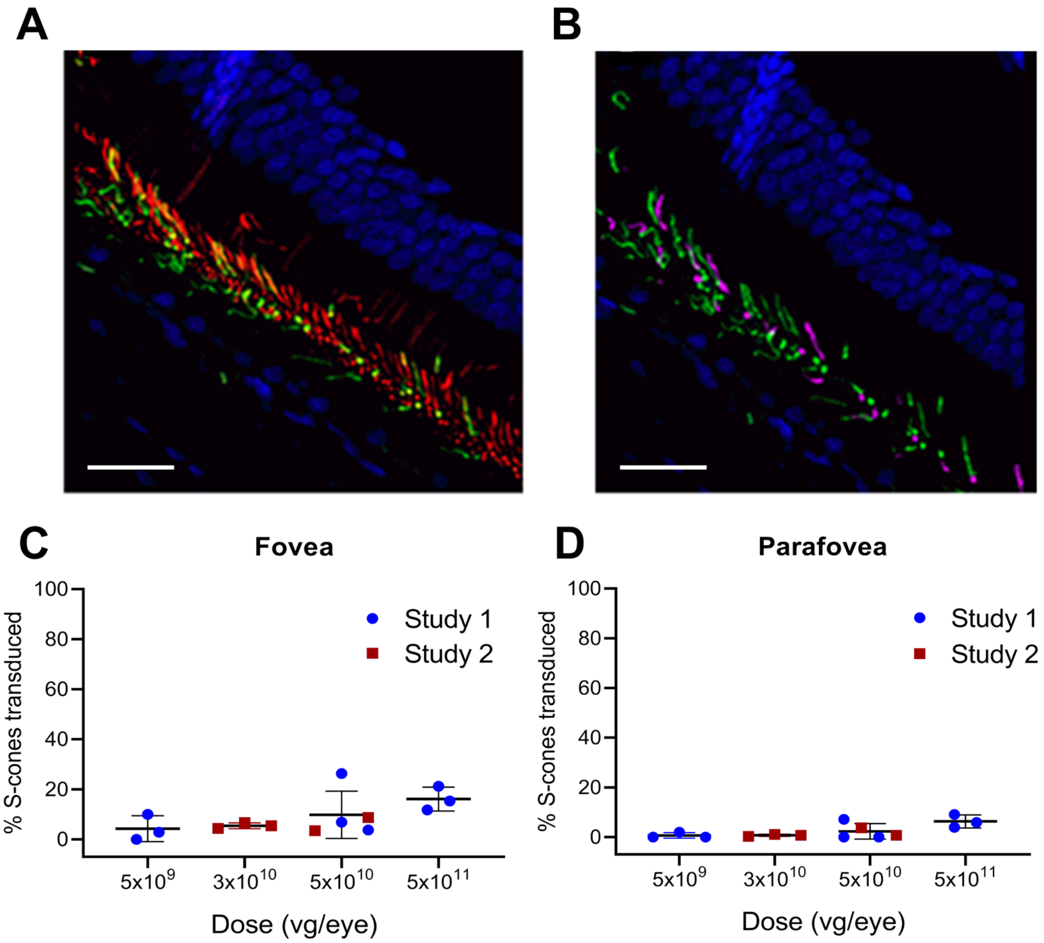

Figure S4. Localization of human L-opsin.myc in NHP S-cones. ADVM-062.myc was IVT dosed at 5×10^9 vg/eye. Colocalization of L-opsin.myc with pan-cone marker arrestin (**A**).
Colocalization of L-opsin.myc *(Green)* with S-cone marker S-opsin (**B**).
Blue: DAPI, *nuclei. Red:* cone arrestin, *Green*: hOPN1LW.myc, *Purple:* S-opsin. Scale bars represent 100µm. Percentage of L-opsin.myc positive S-cones fovea (**C**) and parafovea (**D**). S-cones were identified using S-opsin immunofluorescence. The percentage of human L-opsin.myc/S-opsin double-positive cones was evaluated in series of sections cut through the principal axis of fovea along the fovea-optic disk axis to periphery. Due to the higher concentration of S-cones to fovea periphery, L-opsin.myc-positive S-cones were evaluated in series of sections cut from central fovea to periphery, with peak cone values in the individual animals shown. The dose dependent frequency of L-opsin.myc positive cones was evaluated in animals administered ADVM-062 at 5×10^9 (n=3 animals, 3 eyes), 3×10^{10} (n=3 animals, 3 eyes), 5×10^{10} (n=5 animals, 5 eyes) or 5×10^{11} (n=3 animals, 3 eyes) vg/eye.

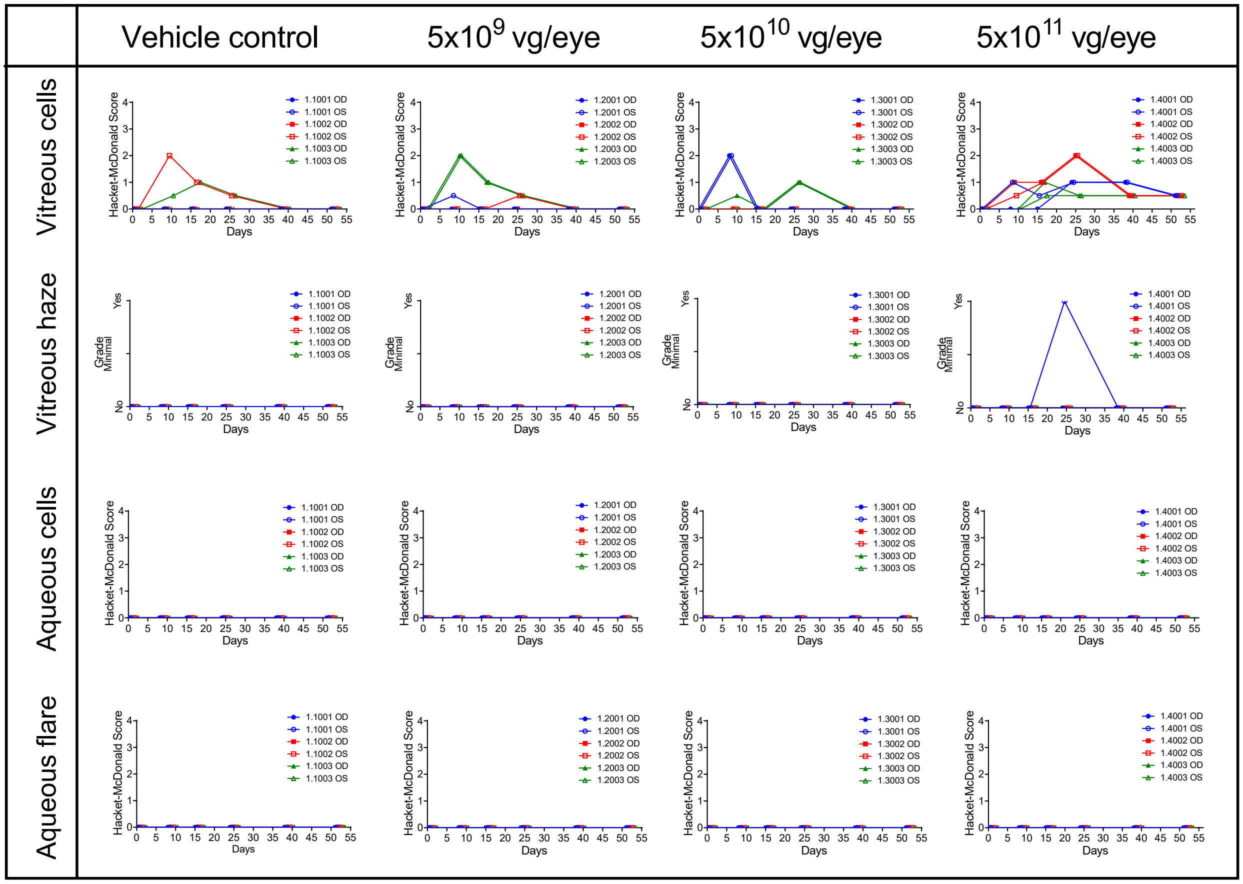

Figure S5. Ocular inflammation findings in the NHPs administered vehicle or ADVM-062.myc at 5×10^{9}, 5×10^{10} and 5×10^{11} vg/eye. All treatment groups: n =3 animals, 6 eyes. Vitreous cell infiltrates, and vitreous haze were observed and showed dose-dependency on ADVM-062.myc.

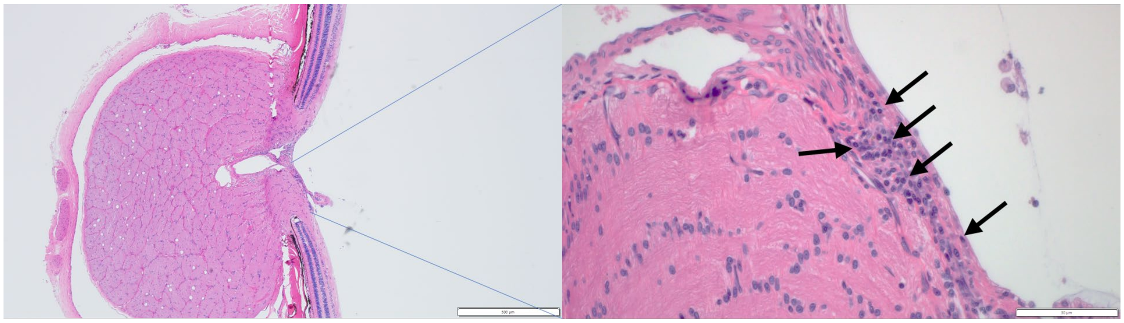

Figure S6. ADVM-062-related microscopic findings of minimal mononuclear infiltrates within the superficial optic disc were observed in the eyes administered highest dose of 3×10^{11} vg/eye (GLP toxicology study, Group 4; Male 4001, OD). Scale bars represent 500μm (left panel) and 50μm (right panel).

Supplemental tables.

Table S1. Probes for in situ hybridization

<table>
<tr><th>Probe Name</th><th>Probe Symbol</th><th>Region of ADVM-062 vector genome sequence complementary to ISH probes (sense)</th></tr>
<tr><td>Homo sapiens L-opsin expression cassette DNA with no cross reactivity against cynomolgus genes</td><td>BA-Hs-L-opsin-No-XMfaSs-3ZZ-st-sense</td><td rowspan="2">TAAGATACATTGATGAGTTTGGACAAACCACAACTAGAATGCAGTGAAAAAAATGCTTTATTTGTGAAATTTGTGATGCTATTGCTTTATTTGTAACCATTATAAGCTGCAATAAACAAGTTAACAACAACAATTGCATTCATTTTATGTTT</td></tr>
<tr><td>Homo sapiens L-opsin transgene mRNA and expression cassette DNA with no cross reactivity against cynomolgus genes</td><td>BA-Hs-L-opsin-No-XMfaSs-3ZZ-st</td></tr>
</table>

Table S2. Dose-ranging study design

Study	Group No.	Animal No.	Treatment	Dose /volume per eye	Dose delivery	Assessments
1	1	1.1001[a]	OU:Vehicle Control	50 µL	Test articles delivered with injections directed towards the mid-vitreous	Ophthalmic Examinations, IOP at pre-treatment and Days 3, 15, and during Weeks 4, 6 and 8.<br><br>ERG and OCT at pre-treatment and during Weeks 4 and 8.<br><br>Human L-opsin.myc IF [b] (OD)
		1.1002[a]				
		1.1003[a]				
	2	1.2001	OU: ADVM-062.myc	5×10^{9}/50 µL		
		1.2002[a]				
		1.2003[a]				
	3	1.3001	OU:ADVM-062.myc	5×10^{10}/50 µL		
		1.3002[a]				
		1.3003				
	4	1.4001[a]	OU:ADVM-062.myc	5×10^{11}/50 µL		
		1.4002				
		1.4003				
2	1	2.2001	OU:ADVM-062.myc	3×10^{10}/50 µL	Test articles delivered with injections directed towards the posterior pole of the eye	Ophthalmic Examinations, OCT and IOP at pre-treatment and Days 7, 14, 28, 42, & 56<br><br>ERG: pre-treatment and Day 55. Human L-opsin.myc IF [b] (OS)
		2.2002				
		2.2003				
	2	2.3001	OD:ADVM-062	5×10^{10}/50 µL		
		2.3002	OS: ADVM-062.myc			

OD = oculus dexter (right eye), OS = oculus sinister (left eye); OU= oculus uterque (both eyes)
[a] Animals that were seropositive for serum neutralizing antibodies prestudy
[b] Human L-opsin.myc IF designates tissue analyzed for localization of human L-opsin.myc transgene and percentage of transgene-positive cones by myc-immunofluorescence

Table S3. GLP toxicology study design

Group	Animal	Treatment	Dose/volume per eye	Assessments
1	1001	OU:Vehicle Control	50 µl	Ophthalmic Examinations and tonometry at pre-treatment and Days 3,8,15,21,26,37,51,65,79,98; ERG and OCT at pre-treatment and on Weeks 4,12. Day 98 – tissue collection
	1002			
2	2001[a]	OU:ADVM-062	5×10^{10} vg/50µL	Ophthalmic Examinations and tonometry at pre-treatment and Days 3,8,15,21,26,37,51,65,79,98; ERG and OCT at pre-treatment and on Weeks 4,12. Day 98 – tissue collection
	2002			
	2003			
3	3001	OU:ADVM-062	1×10^{11} vg/50µL	Ophthalmic Examinations and tonometry at pre-treatment and Days 3,8,15,21,26,37,51,65,79,98; ERG and OCT at pre-treatment and on Weeks 4,12. Day 98 – tissue collection
	3002			
	3003			
4	4001	OU:ADVM-062	3×10^{11} vg/50µL	Ophthalmic Examinations and tonometry at pre-treatment and Days 3,8,15,21,26,37,51,65,79,98; ERG and OCT at pre-treatment and on Weeks 4,12. Day 98 – tissue collection
	4002			
	4003			

[a] Unscheduled test-article-unrelated euthanasia on Day 24.

Table S4. List of Antibodies for Immunofluorescence

Antibody (clone, for monoclonal antibodies)	Vendor (catalog number)	Working Concentration
Calbindin (EP3478)	Abcam (ab108404)	1 µg/mL
Cone Arrestin	Milipore Sigma (AB15282)	2 µg/mL
Peanut agglutinin PNA	Vector Laboratories (RL-1072-5)	5 µg/mL
S-Opsin	Santa Cruz Biotech (SC-14363)	Diluted Stock: 1/500 uL
L/M Opsin	Santa Cruz Biotech (SC-22117)	0.2 µg/mL
MYC-tag	Abcam (ab172)	0.4 µg/mL
Rhodopsin (4D2)	Millipore-Sigma (MABN15)	0.4 µg/mL
Long wavelength-sensitive opsin antibody	Biorbyt (orb182458)	1 µg/mL
GFP	Abcam (ab13970)	10 µg/mL
